# The costs of ideological prosociality: Analyses of the European Social Survey from 2002 to 2018 find negative relationships between endorsing universalistic values and well‐being and social capital

**DOI:** 10.1111/aphw.12385

**Published:** 2022-07-20

**Authors:** John B. Nezlek

**Affiliations:** ^1^ Center for Climate Action and Social Transformations, Institute of Psychology SWPS University of Social Sciences and Humanities Warsaw Poland; ^2^ Department of Psychological Sciences College of William & Mary Williamsburg Virginia USA

**Keywords:** European Social Survey, prosociality, social capital, well‐being

## Abstract

Traditionally, prosociality has been conceptualized in terms of the interpersonal domain, for example, helping behavior. Nevertheless, people can be prosocial in terms of ideological domains, for example, social policies they support. The present study examined the utility of distinguishing interpersonal and ideological prosocial values as predictors of well‐being and social capital. Data from nine European Social Surveys were combined. The *Universalism* and *Benevolence* values of Schwartz's basic human values were treated as measures of ideological and interpersonal prosocial values. Relationships between *Universalism* and *Benevolence* and well‐being and social capital were examined with multilevel models, persons nested with rounds, rounds nested within countries. Respondent sex, age, and education were included as covariates. These analyses found that *Benevolence* was positively related to satisfaction with life and happiness, whereas *Universalism* was negatively related to satisfaction with life and happiness. Although endorsing both values was positively related to attitudinal measures of social capital (e.g., people can be trusted), *Universalism* was negatively related to self‐reports of social activity, whereas *Benevolence* was either positively related or unrelated to self‐reports of social activity. Being ideologically prosocial appears to be associated with reduced well‐being. Future research is needed to explain the mechanisms responsible for this relationship.

## INTRODUCTION

Recently, Nezlek (2022a, 2022b) argued that prosociality can be thought of in terms of two distinct types: Ideological and interpersonal. Interpersonal prosociality refers to people's thoughts, beliefs, and behaviors that concern or are intended to benefit people directly, such as helping and providing social support. In contrast, ideological prosociality refers to people's thoughts, beliefs, and behaviors that concern or are intended to benefit others collectively, such as a concern for human rights, social equality, and environmental quality. This paper concerns how relationships between endorsing prosocial values and well‐being and social capital vary as a function of the type of prosociality being considered.

Previous research on prosociality has considered prosociality primarily, if not exclusively, in terms of what is discussed in this paper as interpersonal prosociality. As explained below and consistent with previous research, the present study was guided by the expectation that endorsing interpersonal prosocial values would be positively related to well‐being and by extension, would be positively related to social capital. In contrast, I expected that endorsing ideological prosocial social values would be negatively related to well‐being but would be positively related to social capital.

This paper examines relationships between well‐being and the endorsement of prosocial values, within the context of large, cross‐national surveys conducted over two decades, the European Social Survey (ESS). Since 2002, the ESS has been conducted every other year, with the exception of 2020, when the survey was suspended due to the COVID‐19 pandemic. In each round of the ESS, a measure of personal values developed by Schwartz and colleagues has been administered. As explained below, Schwartz's measure of *Universalism* was treated as a measure of ideological prosocial values, and Schwartz's measure of *Benevolence* was treated as a measure of interpersonal prosocial values.

### Two types of prosociality

Although ideological and interpersonal prosociality are similar (both refer to benefiting others), as discussed by Nezlek (2022a), they differ in two important ways. First, interpersonal prosociality typically concerns benefitting individuals, whereas ideological prosociality concerns benefitting collectives of individuals or society itself. Helping someone to do something or providing social support benefits a specific person, whereas working for a pro‐environmental organization can be intended to create changes that will benefit everyone. Second, most interpersonal prosocial actions directly benefit a person or persons. For example, Susan helps Jason pick up bags of groceries that have fallen to the ground. The help is immediate and visible to both parties.

In contrast, ideological prosociality typically concerns or involves indirect actions or benefits. For example, Jason may participate in a rally in the hope of changing the policies of his society so that LGBT citizens enjoy the same rights and privileges as non‐LGBT citizens. The existence of the rally may influence public opinion or the opinions of legislators, but the outcome of Jason's actions are far removed from the actions themselves. Moreover, the differences between interpersonal and ideological prosociality mean that individual differences in the two may not be strongly related. For example, someone high in interpersonal prosociality may help a person recover his groceries, while this same person may be low in ideological prosociality and oppose equal rights for LGBT citizens.

### Interpersonal prosociality

Much of the research on prosociality has examined prosociality in terms of what I term interpersonal prosociality, and much of this research has defined prosociality in terms of helping behavior and other positive interpersonal behaviors. For example, when developing a new measure of prosocial intentions, Baumsteiger and Siegel (2019) defined prosociality in terms of helping behavior. A widely used measure of prosociality, the Prosocial Personality Battery (Penner et al., 1995) has seven subscales, each of which measures individually situated prosociality. Another widely used measure of prosociality, Caprara et al. (2005), consists of 16 items, which refer to “one of four types of actions, namely, sharing, helping, taking care of, and feeling emphatic with others and their needs or requests.”

It was worth noting that the values underlying interpersonal prosociality have not received much attention. For many, prosocial behavior is not discussed as a manifestation of prosociality; prosociality is prosocial behavior. A good example of this tendency is a review by Thielmann et al. (2020). In this paper, which is presented as a review of relationships between prosociality and personality, prosociality is defined and discussed, solely and exclusively, in terms of behavior in economic games. The possibility that behaviors in these economic games are manifestations of values, motives, and so forth is not considered.

### Ideological prosociality

Although what I describe as ideologically based prosociality may not have been studied as such, general constructs similar to it have been discussed previously. For example, in their discussion of Social Dominance Orientation (SDO), Ho et al. (2012) suggested that SDO “… is related to the endorsement of a broad spectrum of group‐relevant social ideologies,” “… is related to attitudes toward group‐relevant social policies” (p. 2). Similarly, in a review of vegetarianism, Ruby (2012) concluded that: “Broadly speaking, Western vegetarians tend to be liberal in their political views, place emphasis on environmental protection, equality, and social justice, and oppose hierarchy, authoritarianism, capital punishment, and violence in general” (p. 146). Also, the UN Millennium Goals (UN, 2000) include community of life, ecological integrity, social and economic justice, and democracy, nonviolence, and peace, which represent the gist of ideological prosociality.

Ideological prosocial values are manifested in the holding of attitudes and beliefs and in engaging in behaviors that are not directly intended to benefit specific individuals but are intended to benefit members of collectives or groups. Such collectives may be large (society in general) or limited (members of social minorities). Moreover, the intended beneficiaries do not need to be identified on an individual basis. For example, a person who supports gay rights supports rights for all gays, not just the people he or she knows personally. In contrast, studies of interpersonal prosociality tend to concern beneficiaries who are identified, for example, someone who has been or might be helped.

### Prosociality in the ESS

Similar to most large‐scale cross‐national surveys, the ESS was not intended to study prosociality per se. Nevertheless, respondents in all the ESS rounds answered questions about their values, and these data provided a basis for measuring prosocial values. According to Schwartz (1992), values are trans‐situational goals that serve as guiding principles and that underlie and can help to explain people's decision making, attitudes, and behaviors, and the ESS has measured 21 values proposed by Schwartz (2003, pp. 311–314).

Some of these 21 values can be combined to form two factors, which correspond to the present constructs of ideological and interpersonal prosociality. One factor is *Universalism*, which is defined as “Understanding, appreciation, tolerance, and protection for the welfare of all people and for nature.” Another factor is *Benevolence*, which is defined as the “Preservation and enhancement of the welfare of people with whom one is in frequent personal contact.” The prosocial nature of these two sets of values is also suggested by the fact that together, they comprise a higher order factor labeled *Transcendence*, “… values that emphasize concern for the welfare and interests of others” (e.g., Cieciuch et al., 2015, p. 43),

As mentioned previously, for present purposes, *Universalism* was treated as a measure of ideological prosocial values, and *Benevolence* was treated as a measure of interpersonal prosocial values. Using these two values as measures of ideological and interpersonal prosociality is considered in more detail in the discussion.

### Relationships between well‐being and prosocial values

The ESS administers three widely used measures of individual‐level well‐being: Satisfaction with life, happiness, and self‐reported health, and the present paper concerns relationships between these three measures and prosocial values. The logic of the present study is that values are the foundations of prosociality, and so analyses between well‐being and values can provide a solid basis for making inferences about relationships between prosociality and well‐being.

Most of the existing research on prosociality has concerned interpersonal prosociality, and Hui et al. (2020) conducted a meta‐analysis of research on relationships between prosociality and well‐being that included 201 samples and 198,213 individuals. Although there were some caveats and possible moderators, the general conclusion of this meta‐analysis was that prosociality is positively related to well‐being, and well‐being was defined in ways that are similar to how it is measured in the ESS. Virtually all of the studies Hui et al. examined what I have described as interpersonal prosociality (e.g., helping and volunteering).

Given the newness of ideological prosociality, there is not an established empirical base that can provide a context for understanding relationships between ideological prosociality and well‐being. Nevertheless, there are reasons to believe that ideological prosociality should be negatively related to well‐being. For example, vegetarians who tend to endorse more prosocial attitudes and beliefs than omnivores (Ruby, 2012) tend to have lower well‐being than omnivores (Nezlek & Forestell, 2020). A recent study in Poland compared the psychological distress of members of NGOs with the general population (Cypryańska, 2020). Based on scores on the K10 (Kessler et al., 2002), a measure of distress, Cypryańska found that 25.8% of members of NGOs were distressed, whereas only 8.4% of a comparable sample of the general public were distressed.

Although it may seem that ideological prosociality should be positively related to well‐being (e.g., ideologically prosocial people have positive goals and aspirations), in terms of the state of affairs in the world, there are reasons to expect the opposite. Social equality is part of ideological prosociality; yet social inequality has been increasing worldwide, and people's perceptions of inequality have been found to be negatively related to their well‐being (Delhey & Dragolov, 2013). Being concerned about the effects of climate change is part of ideological prosociality; yet, the quality of the natural environment is deteriorating, worldwide, and well‐being has been found to be negatively related to how serious people perceive environmental problems to be (Rocha et al., 2012). Advocating nonviolent solutions to problems is part of ideological prosociality, but in many parts of the world, there seems to be a near‐constant state of war. Rather than serving as a basis for enhanced well‐being reflecting motivations for making positive changes in the world, an ideological prosocial orientation may serve as a basis for despair, disillusionment, or a lack of hope.

In terms of the ESS per se, analyses of Round 3 (Sortheix & Lönnqvist, 2014) and Rounds 1, 2, and 3 (Sortheix & Schwartz, 2017) found that *Benevolence*, but not *Universalism*, was positively related to life satisfaction. Note that in both of these studies, values were analyzed separately so the covariance between *Universalism* and *Benevolence* was not taken into account.

### Social capital

Social capital has figured prominently in discussions of well‐being for the past few decades. The reason for this is that although social capital is not a measure of well‐being per se, it is presumed to enable or promote well‐being (Helliwell & Putnam, 2004). Among other aspects, social capital includes trust in others, and participation in social, political, and civic life (Putnam, 2000).

Since its beginning, the ESS has measured social capital. Although the individual items that have been included have varied somewhat across rounds, a core of items has been administered in all rounds, and the present paper examines relationships between prosociality and measures of social capital that have been administered in all rounds of the ESS. These measures include trust in others, trust in institutions, and social and civic participation. The specific items selected for analysis are described in the methods section.

The general expectation was that both measures of prosocial values would be positively related to measures of social capital that directly concerned relationships with or perceptions of others: Trust in others and social participation. A foundational element of prosociality is a positive attitude or view of other people, and such relationships represent a manifestation of this foundational element. In contrast, for measures of social capital that did not concern other people directly (e.g., civic participation), existing research and theory did not provide much guidance. Ideological prosociality might be manifested in activities that do not directly involve personal contact, and so ideological prosociality might be related to civic participation whereas interpersonal prosociality, which does not include impersonal activities, may not be related to civic participation.

## METHODS

Relationships between endorsing prosocial values and well‐being and social capital were examined using data collected in the ESS, from its inception in 2002 to the most recent survey, conducted in 2018. Across these nine surveys, 38 countries participated in at least one survey. The mean number of surveys in which a country participated was 6.0, 21 countries (53%) participated in six or more surveys, and 15 countries participated in all nine surveys. A summary of which countries participated in each of the nine surveys is available from the ESS (ESS, 2021a). The ESS summary indicates that Italy participated in Round 2 and Romania participated in Round 3; however, these data are not included in the present analyses because the sample design (and weights) for these samples had not been approved by the ESS. The content of all rounds of the survey was approved by the ESS ERIC Research Ethics Board, and informed consent was obtained in all rounds.

### Sample size

Across all rounds of the ESS, 430,870 people participated. The value items were not included in Round 1 in Italy (*n* = 1207) and in Luxembourg (*n* = 1559), and some individuals did not answer the values questions, so the maximum number of respondents in any analysis was 417,724. The number of these 417,724 respondents who answered each question is presented in Table 1, and the number of cases included in each analysis is provided in Tables 2 and 3. The sample consisted of 53.9% women. A summary of the countries in which the values questions were asked in each round of the ESS is presented in the supplemental materials.

**TABLE 1:** Means and variance estimates at each level of analysis for measures of values, well‐being, social capital, and covariates

	*n*	Mean	Person	Round	Country
*Universalism*	417,724	.52	.396	.005	.023
*Benevolence*	416,908	.61	.416	.007	.043
Life satisfaction	415,650	6.48	4.95	.067	.723
Happiness	415,175	6.94	3.81	.049	.423
Self‐rated health	417,199	3.66	.776	.003	.067
People can be trusted	416,314	4.49	5.44	.035	.643
People are fair	413,899	5.12	4.97	.022	.647
People are helpful	415,591	4.42	5.10	.048	.568
Often meet others	415,434	4.81	2.40	.011	.182
Socially active	408,767	2.69	.845	.004	.024
Political activity	417,017	.72	1.47	.017	.134
Trust: Country's parliament	406,573	4.10	5.91	.238	.893
Trust: Legal system	406,907	4.71	6.17	.135	1.17
Trust: Police	412,684	5.49	5.79	.127	1.68
Trust: Politicians	409,285	3.09	4.99	.164	.546
Trust: European Parliament	370,717	4.17	6.19	.142	.198
Trust: United Nations	373,849	4.72	6.41	.106	.515
Age	416,100	4.53	3.35	.009	.061
Education	416,790	3.53	3.02	.086	.402

**TABLE 2:** Relationships between prosocial values and well‐being, without and with covariates, and tests of the equality of the coefficients for *universalism* and *benevolence* (*χ* ^2^)

Outcome	n	*Universalism*	*Benevolence*	Sex	Age	Education	*χ* ^2^
Life satisfaction	414,866	−.158	.086				46.47
	412,344	−.102	.122	.007^ns^	−.120	.100	112.34
Happiness	414,400	−.121	.126				47.78
	411,895	−.047	.168	.013^ns^	−.133	.090	92.43
Self‐rated health	416,395	−.138	−.069				47.71
	413,852	−.039	−.010**	−.051	−.194	.064	5.08*

*Note*: All coefficients significant at *p* < .0001, except those accompanied by ns, which were not significant, and **, which were significant at *p* < .01. All *χ* ^2^ were significant at *p* < .0001, except the *χ* ^2^ accompanied by *, which was significant at *p* = .02.

**TABLE 3:** Relationships between prosocial values and social capital without and with covariates and tests of the equality of the coefficients for *Universalism* and *Benevolence* (*χ* ^2^)

Outcome	*n*	*Universalism*	*Benevolence*	Sex	Age	Education	*χ* ^2^
People can be trusted	415,527	.177	.111				<1
	412,993	.171	.139	−.053*	−.029^ns^	.149	<1
People are fair	413,137	.183	.163				<1
	410,652	.161	.168	.050**	−.002^ns^	.103	<1
People are helpful	414,817	.074*	.136**				<1
	412,297	.061	.123	.057	−.014^ns^	.049	1.14^ns^
Often meet others	414,650	−.221	.060*				345.4
	412,135	−.098	.115	−.037*	−.216	−.004^ns^	200.3
Socially active	408,049	−.107	−.004^ns^				67.43
	405,604	−.087	.017^ns^	−.027**	−.042	.055	185.2
Political activity	416,216	.186	.043				21.20
	413,674	.170	.069	−.058	−.007^ns^	.152	32.09

*Note*: All coefficients significant at *p* < .0001, except those accompanied by ns, which were not significant, * which were significant at *p* < .05, and ** which were significant at *p* < .01.

### Measures

Detailed descriptions of all measures are available from the ESS (ESS, 2021b). For the convenience of readers, brief descriptions of the measures used in the present study and response scales for each of these measures are presented below.

#### Values

Values were measured using the 21 items proposed by Schwartz (2001). For each item, participants indicated the extent to which a certain statement described them using a 6‐point scale with endpoints labeled 1 = *very much like me* and 6 = *not like me at all*. Ideological prosocial values were conceptualized as the mean response to the three items that Schwartz and colleagues describe as measures of *Universalism*: Understanding different people, treating people equally and supporting equal opportunities for everyone, and caring for nature and the environment. Interpersonal prosocial values were conceptualized as the mean response to the two items that Schwartz and colleagues describe as measures of *Benevolence*: Helping people and caring for others' well‐being and being loyal to friends and devoted to people close to you.

Prior to analysis, responses to the 21 items were reverse‐scored so that higher numbers indicated stronger endorsement of a statement. As recommended by Schwartz (2001), individual differences in the use of the response scale were controlled by calculating the mean response to the 21 values, and then subtracting this mean from the response to each value. Using Mplus (Muthén & Muthén, 2017) to account for the multilevel nature of the data, the estimated correlation between the nonipsatized scores of *Universalism* and *Benevolence* was .33 (*p* < .001), whereas it was .11 for the ipsatized scores (*p* < .001).

#### Well‐being

Well‐being was measured with three items: Life satisfaction, 0 = *extremely dissatisfied*, 10 = *extremely satisfied*; Happiness, 0 = *extremely unhappy*, 10 = *extremely happy*; and subjective health, 1 = *very good*, 5 = *very bad*. Ratings of subjective health were reversed‐scored prior to analysis.

#### Social capital

Interpersonal social capital was measured with five items. Three items concerned attitudes toward people in general: Most people can be trusted or you cannot be too careful; Most people try to take advantage of you, or try to be fair; and Most of the time people are helpful or mostly looking out for themselves. Responses were made using 0–10 positively valent scales. Two items concerned social involvement: How often do you socially meet with friends, relatives, or colleagues; and How often do you take part in social activities compared with others of the same age. The response scale for the first of these was a 1–7 scale, and the response scale for the second was a 1–5 scale.

Institutional trust was measured for six institutions: home country's parliament, legal system, police, politicians, European Union, and the United Nations. Responses were made on a 0–10 scale (0 = *not trust all*, 10 = *complete trust*). Political involvement was measured with seven yes/no items referring to political activity over the last 12 months: contacted politician or government official, worked for a political party or action group, worked in another organization or association, worn or displayed campaign badge/sticker, signed petition, took part in a lawful public demonstration, and boycotted certain products. As discussed below, the results of the analyses of these dichotomous measures were very similar, and so an overall measure labeled “Political activity” was created, which consisted of the sums of the individual items, adjusted for the number of nonresponses to individual items.

#### Covariates

To control relationships between well‐being and prosocial values for individual differences in sociodemographic characteristics, the analyses included the following covariates: respondent gender, represented by a contrast‐coded variable (1 = women, −1 = men), age, and education. Education was measured using the ISCED codes (UNESCO, 2011), which range from 1 to 7.^1^ Age ranged from 13 to 80, and to reduce the influence that differences in the variances of covariates might have on estimates of coefficients, age was divided by 10 prior to analysis.

#### Supplemental materials

The supplemental materials mentioned in this paper are available via the Open Science Framework: https://osf.io/cdxhs/?view_only=52b12bf35a874e17a5482696dca99e8f.

## RESULTS

### Overview of analyses

The data were organized as a three‐level data structure in which persons were nested within rounds which were nested within countries, and the data were analyzed using the program HLM (Raudenbush et al., 2019). The analyses followed guidelines offered by Nezlek (2010), and observations were weighted to adjust for sample characteristics. The ESS provides weights for the person‐ and country‐levels that are specific to each round. In the present analyses, person‐level observations were weighted by the ESS variable “pspweight.” Country‐level observations within each round were weighted by the ESS variable “pweight.” Using weights in the ESS is discussed by Kaminska and Lynn (2017).^2^

There were three sets of analyses: (1) Unconditional models that were used to generate descriptive statistics, (2) models in which measures of well‐being were regressed on the two measures of prosociality, and (3) models in which measures of well‐being and social capital were regressed on the two measures of prosocial values and the three demographic variables as covariates. In all analyses, random effects for each coefficient and the covariances between these random effects were estimated. Moreover, in each set of analyses, significance tests for coefficients representing prosocial values were corrected for family‐wise error (defined as coefficients for the same predictor across outcomes) using the false discovery rate technique developed by Benjamini and Hochberg (1995). In all cases, coefficients for prosocial values that were significant at *p* < .05 uncorrected remained significant after correction.

### Basic, unconditional model and descriptive statistics

Each measure was analyzed with an unconditional model, that is, no predictors at any level of analysis. Such analyses provide the basic descriptive statistics for multilevel data, the mean and the variance estimates at each level of analysis. In the model presented below, there are *i* individuals nested within *j* rounds nested within *k* countries. The variance of *e*_ijk_ is the person‐level variance, the variance of *r*_0jk_ is the round‐level variance, and the variance of *μ*_00k_ is the country‐level variance. The results of these analyses are summarized in Table 1.

Person‐level: *y_ijk_* = *π_0jk_* + *e_ijk_*
Round level: *π_0jk_* = *β_00k_* + *r_0jk_*
Country level: *β_00k_* = γ_000_ + *μ_00k_*

Although these descriptive statistics do not test hypotheses per se, they are informative. First, for all measures, the majority of the total variance (over 85%) was at the person level. People varied more than rounds and countries varied. This suggested that analyses at the person‐level (the focus of this study) could be productive. If there had not been much variance at the person‐level this would have suggested that it would be difficult to model person‐level relationships.

Second, none of the means were so low or so high to suggest floor or ceiling effects. On average, people were happy, satisfied with their lives, and in good health, they had personal social capital, and they trusted institutions. The range for *Universalism* was −3.57 to 3.75, and it was −3.78 to 4.17 for *Benevolence*.

In addition to these summary statistics, correlations among all measures at each level of analysis were estimated using Mplus. These correlations are presented in a table in the supplemental materials.

### Relationships between endorsing prosocial values and well‐being

Relationships between endorsing prosocial values and well‐being were examined by regressing measures of well‐being onto ideological and interpersonal prosocial values, that is, *Universalism* and *Benevolence*. Measures of prosocial values were entered group‐mean centered and as randomly varying. The model is below.

Person‐level: *y*_ijk_ = *π_0jk_* + *π_1jk_* * (*Universalism*) + *π_2jk_* * (*Benevolence*) + *e_ijk_*
Round level: *π_0jk_* = *β_00k_* + *r_0jk_* (Intercept)

$$\pi_{\text{1jk}}=\beta_{10k}+r_{\text{1jk}}\;\left(\text{Universalism}\right)$$

$$\pi_{\text{2jk}}=\beta_{20k}+r_{\text{2jk}}\;\left(\text{Benevolence}\right)$$

Country level: *β_00k_* = *γ_000_* + *μ_00k_* (Intercept)

$$\beta_{10k}=\gamma_{100}+\mu_{50k}\;\left(\text{Universalism}\right)$$

$$\beta_{20k}=\gamma_{200}+\mu_{50k}\;\left(\text{Benevolence}\right)$$

The results of these analyses are summarized in Table 2. As can be seen from this summary, satisfaction with life and happiness were positively related to interpersonal prosocial values (*Benevolence)*, whereas they were negatively related to ideological prosocial values (*Universalism)*. Unexpectedly, self‐reported health was negatively related to both *Universalism* and *Benevolence*. Moreover, the equality of the coefficients for *Universalism* and *Benevolence* was examined with a test of the effect on the model fit of constraining the coefficients to be equal (Nezlek, 2010, pp. 328–330). To test a constraint, one compares the goodness of fit for two models: one with the constraint and one without. The difference between the two goodness of fit measures is distributed as a chi‐square with degrees of freedom corresponding to the number of parameters involved in the constraint. These analyses, the results of which are also summarized in Table 2, found that the coefficients for *Universalism* and *Benevolence* were significantly different in the analyses of all three measures of well‐being.

To control for individual differences in demographic characteristics, respondent sex, age, and education were added as covariates to the model above. Age and education were entered group‐mean centered, and the sex‐contrast variable was entered uncentered. All slopes at level‐1 and level‐2 were modeled as randomly varying. The model is below, and in the interests of brevity, the level‐2 and level‐3 equations are not presented.

Person‐level:

$$y_{\mathrm{ijk}}=\pi_{\text{0jk}}+\pi_{\text{1jk}}*\left(\text{Universalism}\right)+\pi_{\text{2jk}}*\left(\text{Benevolence}\right)+\pi_{\text{3jk}}*\left(\mathrm{Sex}\right)+\pi_{\text{4jk}}*\left(\mathrm{Age}\right)+\pi_{\text{5jk}}*\left(\text{Education}\right)+e_{\mathrm{ijk}}$$

The results of these analyses are summarized in Table 2. Similar to the results of the analyses without the covariates, satisfaction with life and happiness were positively related to interpersonal prosocial values (*Benevolence)*, whereas they were negatively related to ideological prosocial values (*Universalism)*, and *self‐reported health* was negatively related to both *Universalism* and *Benevolence*. The coefficients for *Universalism* in these analyses were smaller than they were in the original analyses, but as before, tests of the equality of the coefficients for *Universalism* and *Benevolence* found that they were significantly different in the analyses of all three measures of well‐being. Estimates of the random effects for these analyses are presented in a table in the supplemental materials.

To provide a context for understanding the differences in the size of the coefficients between the two sets of analyses, the covariates were regressed onto both measures of prosocial values. A model with age as the outcome and *Universalism* and *Benevolence* as predictors found that both age was positively related to *Universalism* and *Benevolence* (*Universalism*, *γ*_100_ = .512, *p* < .0001; *Benevolence*, *γ*_200_ = .228, *p* < .0001). The slight enhancement effect for *Benevolence* that occurred in the analyses of satisfaction with life and happiness was due to the fact that education was positively related to *Universalism* (*γ*_100_ = .105, *p* < .001), whereas education was negatively related to *Benevolence* (*γ*_200_ = −.083, *p* < .0001). A logistical MLM found that being male was negatively related to both *Universalism* and *Benevolence* (*Universalism*, *γ*_100_ = −.379, *p* < .0001; *Benevolence*, *γ*_200_ = −.243, *p* < .0001).

### Changes across time

On an exploratory basis, I examined if relationships between well‐being and prosocial values varied across time. This was done by creating a variable representing the linear trend across the nine surveys (−4, −3, −2, −1, 0, 1, 2, 3, 4). This trend measure was entered uncentered and randomly varying at level‐2 in the models examining relationships between well‐being and *Universalism* and *Benevolence*, and these analyses included the covariates. The trend effect was not significant (*p* > .12) for *Universalism* and *Benevolence* in the analyses of all three measures of well‐being (absolute value of coefficients for all trends less than .01). These results suggest that relationships between well‐being and prosocial values did not vary across time.

### Relationships between endorsing prosocial values and social capital

Relationships between prosocial values and social capital were examined with models that were the same as those used to examine relationships between prosocial values and well‐being. The results of these analyses, including the analyses of the combined measure of political activity, but not including the analyses of intuitional trust, are summarized in Table 3.^3^

The analyses of attitudes toward people in general (can be trusted, are fair, and are helpful), found that both types of prosocial values were positively related to all three measures, and the coefficients for *Universalism* and *Benevolence* did not differ significantly. In contrast, the analyses of social involvement (meet people, socially active) found that *Universalism* was negatively related to both measures, whereas *Benevolence* was positively related to the first and was unrelated to the second. Moreover, for both measures, the coefficients for *Universalism* and *Benevolence* were significantly different. The analyses of the combined measure of political activities, summarized in Table 3, found positive relationships between both *Universalism* and *Benevolence* and political activity. The relationship between political activity and *Universalism* was stronger than the relationship between political activity and *Benevolence*. Estimates of the random effects for these analyses are presented in a table in the supplemental materials.

The analyses of institutional trust found few significant relationships between trust and prosocial values. *Universalism* was positively related to trust in the European Parliament and the UN (*γ*_100_ = .062 and .075, *p* < .01, *p* < .0001, respectively). In contrast, *Benevolence* was negatively related to trust in politicians and the European Parliament (*γ*_200_ = −.085 and −.061, respectively, *p*s < .001), and was not significantly related to trust in the UN (*γ*_200_ = −.016, *p* > .25). The results of these analyses are summarized in a table in the supplemental materials.

Given the relationships that were found between prosocial values and social capital, analyses were done that controlled relationships between prosocial values and well‐being for individual differences in social capital. With one exception, although some of the coefficients for prosocial values in some of these analyses were smaller than they were in the initial analyses, they were roughly equivalent in magnitude. The one exception to this pattern was the analysis of happiness. When social activity was included as a covariate, the coefficient for *Universalism* decreased to −.017 and was not significant (*p* = .07). Recall that in the original analysis of happiness (with covariates), the coefficient for *Universalism* was −.047 and it was significant (*p* < .0001).

### Controlling for political orientation

The preceding analyses did not control for respondents' political orientation. Although a measure of left–right political orientation was included in the ESS (placement on left–right scale, 0 = left, 10 = right), for various reasons, approximately 14% of respondents who answered the values questions did not answer this question. In some countries, in some waves, up to 40% of responses to this item were missing. MLM analyses use listwise deletion, so including political orientation in an analysis could lead to biased estimates, that is, coefficients based on only people who indicated their political orientation.

Nevertheless, existing research suggests that conservatives tend to be happier than liberals (e.g., Napier & Jost, 2008) and that conservatives tend to be less prosocial than liberals in terms of both *Universalism* and *Benevolence* (e.g., Nezlek, 2022a). Given these relationships, it was important to determine if controlling for political orientation would change estimates of relationships between well‐being and prosocial values, with the understanding that the results of such analyses might be biased. This was done by conducting analyses in which life satisfaction, happiness, and self‐reported health were regressed onto the two measures of prosocial values and political orientation. The results of these analyses support the conclusions based on the presented in Table 2. A table containing a summary of these analyses is available in the supplemental materials.

#### Effect sizes

Although comparisons of residual variances can be used to estimate effect sizes within the multilevel context (similar to ordinary‐least squares analyses), the estimates produced by such comparisons need to be considered cautiously, particularly when there are multiple predictors. See Nezlek (2011, pp. 35–36) for a discussion. With this in mind, I calculated reductions in residual variances for the models with covariates presented in Tables 2 and 3 when compared with null models. These estimates were as follows: Life satisfaction, 2.95%; happiness, 3.78%; self‐reported health, 20.99%; trust in people, 3.35%; fairness of people, 2.28%; helpfulness of people, 1.33%; meeting other, 8.30%; social activity, 3.19%; and political activity, 9.42%.

## DISCUSSION

As expected, endorsing ideologically prosocial values was negatively related to well‐being, whereas endorsing interpersonal prosocial values was positively related to well‐being in terms of happiness and life satisfaction. Although these results met expectations, they raise important questions, and I discuss some of these below.

### Well‐being

It may be informative to consider the present results in terms of the distinction between hedonic and eudaimonic well‐being. Broadly speaking, hedonic well‐being refers to happiness or pleasure, whereas eudaimonic well‐being refers to the extent to which people are living in accordance with their *daimon*, or true self (Waterman, 1993). In terms of the items that were the focus of the present analyses, happiness is perhaps the archetypal measure of hedonic well‐being. Although self‐reported health does not concern happiness or pleasure per se, it is clearly not a measure of eudaimonic well‐being.

In contrast, satisfaction with life may represent a mix of hedonic and eudaimonic well‐being. For example, when discussing differences between life satisfaction and happiness, Kahneman and Deaton (2010, p. 16492) mentioned “the importance of the distinction between the judgments individuals make when they think about their life and the feelings that they experience as they live it.” Along the same lines, Kapteyn et al. (2015) distinguished measures of evaluative well‐being such as life satisfaction from measures of experienced well‐being such as happiness, and in their factor analyses the single item they included that measured eudaimonic well‐being loaded on a life satisfaction factor and not on any of the experienced well‐being factors they found.

The evaluative‐experienced distinction maps onto differences between the results of the analyses of satisfaction with life and happiness that included social activity as a covariate. Including social activity as a covariate in the analysis of happiness rendered the negative coefficient for *Universalism* nonsignificant, whereas social activity (either measure) had virtually no effect on the negative coefficient for *Universalism* in the analysis of satisfaction with life. This difference suggests, as proposed by Kapteyn et al. (2015), that reports of happiness reflect people's immediate circumstances more than reports of life satisfaction do. People who are high in *Universalism* may be less happy than those low in *Universalism* because of the quality of their immediate social environments, but the negative relationship between satisfaction with life and *Universalism* cannot be explained by individual differences in immediate social environments. This suggests that satisfaction with life represents something broader than experienced well‐being, at least as measured by happiness.

As mentioned in the introduction, compared with people low in *Universalism*, people high in *Universalism* may be more likely to believe that society (broadly defined) does not value what they value. Such beliefs may undermine broad‐based measures of well‐being such as satisfaction with life, measures that may reflect eudaimonic well‐being in part. In contrast, being high in *Universalism* may lead to greater social distance, which is reflected in lower happiness, that is, immediate experience. Resolving such issues will take studies (or analyses of existing studies) that have measures of values and of eudaimonic well‐being.

### Social capital

When discussing the present results for social capital, it is useful to consider a distinction discussed by Helliwell and Putnam (2004, p. 1437): “… we need to distinguish among different types of social capital, like the difference between ‘bonding’ social capital—these are links among people who are similar in ethnicity, age, social class, etc.—and ‘bridging’ social capital, which are links that cut across various lines of social cleavage.” Two measures of interpersonal social capital (meeting and socially active) represent bonding social capital, and *Universalism* was negatively related to these measures. The three other measures of interpersonal prosociality, which did not refer specifically to close or distant others, probably represented a blend of bridging and bonding social capital, and both measures of prosocial values were positively related to all three measures.

The bridging‐bonding distinction does not seem directly relevant to the other measures of social capital, that is, trust in institutions and political activity. For the most part, these measures were positively related to *Universalism* and were either unrelated, negatively related, or more weakly related to *Benevolence*. The one exception to this was trust in the police, which was positively related to *Benevolence*. The positive relationships between *Universalism* and trust in the EU and UN may have been due to beliefs that these two institutions are guided by values and principles that are consistent with *Universalism*. These organizations are meant to promote social justice, economic well‐being for all citizens, a healthy environment, and so forth, and the more ideologically prosocial people are the more they might identify with and support these institutions. Verifying this will take research specifically designed to do so.

### How well do *Universalism* and *Benevolence* measure ideological and interpersonal prosociality?

Although *Universalism* and *Benevolence* are measures of prosocial values, they were not intended to be measures of ideological and interpersonal prosociality per se. Moreover, there are only three and two items (respectively) for the two constructs, which may limit their generalizability. Both types of prosociality are meant to be broader constructs that include other components. For example, interpersonal prosociality includes individual differences such as empathy and agreeableness, both of which have been long considered to be part of prosociality. Ideological prosociality may include individual differences such as need for closure, open‐mindedness, and tolerance of ambiguity, all of which have been found to be related to socio‐political attitudes. Clearly, future work needs to examine such possibilities.

Moreover, *Benevolence*, as defined by Schwartz, explicitly concerns people who are close to the respondent, for example, family and friends. *Benevolence* does not include prosocial values as they pertain to unfamiliar others. For example, within the self‐portrait format that Schwartz and colleagues have used, items such as “It is important to him that strangers and people who do know him well can trust him,” and “It is important to her to help strangers and people she does not know well who are in need,” would not be considered to be part of *Benevolence* because they do not refer to close others. Such values are not, and have not been, part of Schwartz's model. Within the proposed framework, such values would be part of interpersonal prosociality, and establishing this requires research that is explicitly designed to do so.

### Why is endorsing ideological prosocial values negatively related to well‐being?

As suggested in the introduction, the negative relationships between *Universalism* (ideologically prosocial values) and well‐being may reflect people's perceptions of the world and its future. People who endorse *Universalistic* values more strongly may be more dissatisfied with the state of the world and more pessimistic about its future than people who endorse *Universalistic* values less strongly. In turn, this dissatisfaction and pessimism results in diminished well‐being.

Some might argue that such a set of relationships would mean that how strongly people endorse *Universalistic* values is not related to well‐being because such relationships reflect some type of combination of values and perceptions/expectations. If constructs such as optimism/pessimism were found to mediate relationships between values and well‐being, this would not invalidate the zero‐order relationships between values and well‐being. Rather, such mediational relationships would help explain the zero‐order relationships. Unfortunately, the ESS does not provide a basis to determine if dissatisfaction with the state of the world and pessimism about its future mediates relationships between *Universalism* and well‐being.

### Limitations and future directions

Among a sample of approximately 400,000 respondents taken from 38 countries over nearly 20 years *Universalism* was found to be negatively related to well‐being, whereas *Benevolence* was found to be positively related. The breadth of this sample leaves little doubt about the validity of these findings, but important questions remain unanswered. Some such questions concern the mechanisms that might explain these relationships. Given that relationships between values and well‐being are at the individual level, such possibilities could be examined using mediational analyses such as those just described. Our understanding of relationships between values and well‐being may also be furthered by examining moderators of these relationships. Such moderation could exist at both the individual‐level (e.g., do relationships between values and well‐being vary as a function of personality) and the country‐level (e.g., do relationships between values and well‐being vary as a function of per capita income). There are many measures that could be mediators and moderators in such analyses. The challenge is to select measures for such analyses on sound theoretical or empirical bases.

There is also the issue of the strengths of these relationships. It is likely that the outcomes measured in the present study are influenced by multiple factors, of which values are only a (perhaps small) part. Nevertheless, as noted by Sortheix and Lönnqvist (2014, p. 295), the present effect sizes are similar to those found in other studies of satisfaction with life. Perhaps stronger effects might be found in analyses of moderation.

The present study examined the importance of distinguishing ideological and interpersonal prosociality, using two values proposed by Schwartz and colleagues. Other values proposed by Schwartz and colleagues may be related to well‐being in ways that complement the present findings. It is also important to note that the ESS measures what are known as “second order factors” within research on Schwartz's model. Each of the individual items in the ESS measures the first‐order factors, which themselves can be measured with three items (Schwartz et al., 2012). Future research using these first‐order factors may provide some insights into the hows and whys of relationships between well‐being and prosocial values.

Finally, the present analyses concerned prosocial values, which are only part of the broader constructs of ideological and interpersonal prosociality. Although values may be foundational elements of prosociality, it cannot be assumed that relationships between well‐being and ideological and interpersonal prosociality will be the same as relationships between well‐being and *Universalism* and *Benevolence*. Determining this will require research that is designed to examine explicitly such relationships. Regardless, the present study provides support for distinguishing ideological and interpersonal prosociality, and in so doing provides a rationale for research examining this distinction.

## CONFLICT OF INTEREST

The authors declare no conflict of interest.

## ETHICS STATEMENT

The content of all rounds of the survey was approved by the ESS ERIC Research Ethics Board, and informed consent was obtained in all rounds.

## Data Availability

All data are available from the European Social Survey https://www.europeansocialsurvey.org/about/.

## ENDNOTES

^1^
For 39 of the 228 combinations of country and round, ISCED codes were not provided, although years of education was. To maximize the number of observations that were included in the analyses, ISCED codes were estimated for observations for which ISCED codes had not been estimated using number of years of education as a basis. How this was done is described in the supplemental materials.

^2^
I am grateful to Olena Kaminska for her help regarding selecting the weights for these analyses.

^3^
The seven yes/no items referring to political activity were analyzed with multilevel logistical regressions that had the same predictors and covariates as the other models. These analyses found that both *Universalism* and *Benevolence* were positively related to all seven measures. Moreover, for four of the seven measures, the coefficients for *Universalism* were stronger than the coefficients for *Benevolence*. The results of the analyses of the individual items are summarized in a table included in the supplemental materials.
